# Rapid Detection of Fatty Acids in Edible Oils Using Vis-NIR Reflectance Spectroscopy with Multivariate Methods

**DOI:** 10.3390/bios11080261

**Published:** 2021-08-03

**Authors:** Ning Su, Fangfang Pan, Liusan Wang, Shizhuang Weng

**Affiliations:** 1 Institute of Intelligent Machines, Hefei Institutes of Physical Science, Chinese Academy of Sciences, Hefei 230031, China; oksuning@mail.ustc.edu.cn; 2 Intelligent Agriculture Engineering Laboratory of Anhui Province, Hefei 230031, China; 3 National Engineering Research Center for Agro-Ecological Big Data Analysis and Application, Anhui University, Hefei 230601, China; ahusei@126.com

**Keywords:** Vis-NIR reflectance spectroscopy, multivariate analysis, fatty acid, edible oil, quality detection

## Abstract

The composition and content of fatty acids are critical indicators to identify the quality of edible oils. This study was undertaken to establish a rapid determination method for quality detection of edible oils based on quantitative analysis of palmitic acid, stearic acid, arachidic acid, and behenic acid. Seven kinds of oils were measured to obtain Vis-NIR spectra. Multivariate methods combined with pretreatment methods were adopted to establish quantitative analysis models for the four fatty acids. The model of support vector machine (SVM) with standard normal variate (SNV) pretreatment showed the best predictive performance for the four fatty acids. For the palmitic acid, the determination coefficient of prediction ($R_{P}^{2}$) was 0.9504 and the root mean square error of prediction ($RMSE_{P}$) was 0.8181. For the stearic acid, $R_{P}^{2}$ and $RMSE_{P}$ were 0.9636 and 0.2965. In the prediction of arachidic acid, $R_{P}^{2}$ and $RMSE_{P}$ were 0.9576 and 0.0577. In the prediction of behenic acid, the $R_{P}^{2}$ and $RMSE_{P}$ were 0.9521 and 0.1486. Furthermore, the effective wavelengths selected by successive projections algorithm (SPA) were useful for establishing simplified prediction models. The results demonstrate that Vis-NIR spectroscopy combined with multivariate methods can provide a rapid and accurate approach for fatty acids detection of edible oils.

## 1. Introduction

The consumption of various edible oils has been increasing due to the growth of population. Edible oil is good of taste and health properties which provides many health beneficial substances including fatty acids, energy, and other essential trace elements [1]. The quality of edible oil is closely related to public health and food safety and the choice of edible oils affects the nutritional balance of the human body [2]. However, the quality of edible oils is frequently subjected to adulteration, contamination, deterioration, and re-use problems in production process [3,4], which greatly affects their edibility. The external features of edible oils are easily tampered by physical and chemical means, it is more difficult to discriminate the quality of edible oils based only on color, smell, and taste [5,6]. Every kind of pure edible oil has a relatively stable composition ratio of fatty acids, and all the above quality problems cause the significant change of the content of fatty acids in terms of the internal composition of edible oils. Therefore, the quantitative analysis of fatty acids would be an effective method to assess the quality of edible oils.

Some studies have been conducted to precisely measure the content of fatty acids for quality assessment of oil products in recent years [7]. Gas chromatography was the preferred analytical method for the determination of fatty acid methyl esters (FAMEs) [8,9,10]. High-performance liquid chromatography was also used for the analysis of fatty acids in biological samples [11]. The nuclear magnetic resonance technology can be used to measure fatty acids in edible oils [12]. In addition, fluorescent spectroscopy was studied as a detection method in oil quality testing [13]. Except fluorescent spectroscopy, these detection methods involve a series of time-consuming experimental preparation, such as extraction, derivatization, and chromatography steps. Meanwhile, hazardous chemicals are utilized in these methods; therefore, skilled analytical technicians are required. Fluorescent spectroscopy is a non-destructive method for discriminating edible oils without pretreatment steps. However, fluorescent spectroscopy depends on bulky and expensive fluorescent devices that severely limits its application in edible oil industry. All of these hamper the widespread applications of these methods. Therefore, a simple, rapid, and economical method is of high demand to detect the fatty acids of edible oils. In recent years, near-infrared spectroscopy was widely applied for oil detection. Near-infrared spectroscopy is an optical detection technique that measures the interaction of infrared radiation with analytes by absorption or reflection. The characteristics of molecular vibration and rotation of analytes can be obtained by inspecting the absorption spectra. For example, the rapid identification of edible oil and swill-cooked dirty oil was studied by employing near-infrared spectroscopy and the sparse representation classification method [14]. Jiang et al., applied a near-infrared spectroscopy system to determine acid values during edible oil stored procedures [15]. However, the near-infrared spectroscopy was generally effective for qualitative analysis, while quantitative analysis based on near-infrared spectroscopy was not ideal in some studies of edible oils [16]. The wavelengths in near-infrared spectrum range mainly reflect the molecular vibration characteristics of objects. The visible spectrum range is a very important supplement for distinguishing the objects based on the basic physical forms. Therefore, the integrated analysis of visible spectrum and near-infrared spectrum can provide more comprehensive spectral information [17]. The visible and near-infrared part of the electromagnetic spectrum includes both the visible (350–780 nm) and near-infrared (780–2500 nm) ranges. However, the application of Vis-NIR reflectance spectroscopy in food composition detection is still restricted to the construction of the accurate data analysis model.

In this study, we presented an efficient and non-destructive method for the fatty acids detection of edible oils using Vis-NIR reflectance spectroscopy. The accurate computational models were constructed based on the Vis-NIR spectra data. The Vis-NIR spectra data were obtained by a hyperspectral spectroradiometer system to give a comprehensive characteristic information of edible oils. The palmitic acid (C16:0), stearic acid (C18:0), arachidic acid (C20:0), and behenic acid (C22:0) were chosen as experimental objects in this paper. Palmitic acid is a saturated long-chain fatty acid which accounts for 4.6–20.0% dominated in edible oils [18]. Stearic acid is a kind of saturated fatty acid with health benefits. It has no effect to increase the plasmatic levels of low-density lipoprotein [19] in contrast to other fatty acids in edible oils. Arachidic acid is kind of a saturated fatty acid with a 20-carbon chain. It can be a chemical messenger released by the muscle that controls the physiological response to the exercise [20]. Behenic acid is a saturated fatty acid that can promote the cholesterol levels in humans [21]. The four fatty acids are commonly found in various vegetable edible oils. They have specific physiological functions for human body and the relatively stable molecular structure resistance to temperature and other environmental factors. Therefore, the contents of the four fatty acids can be used as important references for the quality evaluation of edible oils. The four fatty acid contents were measured by the gas chromatography-mass spectrometry (GC-MS) as standard references for the purpose of model training and validation. The multivariate methods including partial least squares regression (PLSR), support vector machine (SVM) and random forest (RF) were applied with multiple pretreatments of spectra to establish prediction models for content prediction of the four fatty acids. In addition, the successive projections algorithm (SPA), variable importance of projection (VIP) and principal component analysis (PCA) were used for selecting the effective wavelengths to simplify the prediction models. The accurate quantitative analysis of the four fatty acids could provide a critical reference for quality assessment of edible oils.

## 2. Materials and Methods

### 2.1. Oil Samples

In this study, seven kinds of commonly consumed edible oils were randomly collected from local Walmart stores (Hefei, China). There were 93 oils including 15 brands of sesame oil, 15 brands of soybean oil, 11 brands of corn oil, 11 brands of sunflower oil, 13 brands of rapeseed oil, 15 brands of peanut oil, and 13 brands of olive oil obtained in the final sample set. All the oils were firstly stirred evenly for the separation of experimental samples. A piece of oil sample was taken from each of brand edible oils (20 mL/piece). A total of 93 oil samples were first used for Vis-NIR reflectance spectroscopy experiments. After spectra measurement, the four fatty acid contents in each piece of oil sample were analyzed by GC-MS.

### 2.2. Measurement of Vis-NIR Reflectance Spectra of Oil Samples

**Figure 1:**
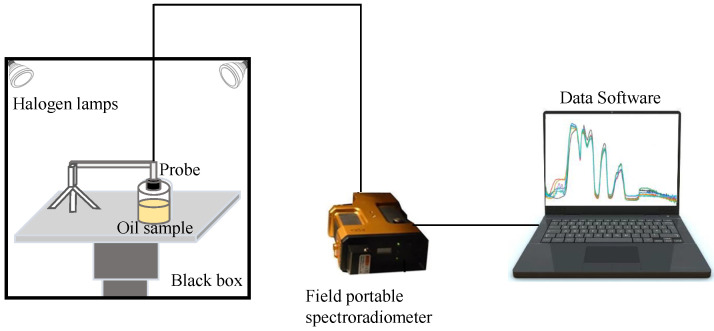
The spectral acquisition system for edible oils.

The Vis-NIR spectra of the 93 oil samples were measured by a field portable spectroradiometer (PSR-3500, Spectral Evolutions, Lawrence, MA, USA). The field portable spectroradiometer is a spectral remote sensing instrument that can achieve the fast and stable measurement of Vis-NIR spectra of objects. Three array detectors (one 512Si detector and two 256InGaAs detectors) were equipped in the hyperspectral instrument to take a accurate measurement of oil samples. The measurement range of the Vis-NIR spectra was 350–2500 nm. The spectral acquisition lens was set to 3 cm away from the oil samples. The optical fiber with field angle of 8 degree was used to measure the Vis-NIR spectra. Each piece of the oil samples was placed in a separated glass beaker (25 mL), as illustrated in Figure 1. For each piece of oil samples, ten Vis-NIR reflectance spectra were recorded with the resolution of 1 nm. To ensure the randomness of the spectrum in each measurement, the 93 oil samples were measured in ten different batches. To be specific, all the oil samples were measured in an independent batch and the process was repeated 10 times. Hence, a total of 930 Vis-NIR reflectance spectra data were measured for the following data analysis.

For each spectrum, there were 2151 data points spreading over all the 350–2500 nm wavelengths. A black box system with fixed light source was constructed for the acquisition Vis-NIR spectra to exclude the interference from external light. In order to eliminate some of the disturbance factors of the black box system, a whiteboard calibration was applied in our experiment of Vis-NIR spectra acquisition [22]. The final Vis-NIR spectra were calibrated based on Equation (Equation (1)) as follows:

(1) $$\begin{array}{cc} R_{mi} & =\frac{DN_{mi}}{DN_{ri}}*R_{ri} \end{array}$$

where $R_{mi}$ is the corrected result of the oil sample and $R_{ri}$ is reflectance the whiteboard. $DN_{mi}$ and $DN_{ri}$ are the original values for the oil samples and the whiteboard, respectively.

### 2.3. Measurement of Four Fatty Acid Contents in Oil Samples

The composition of various fatty acids is an important indicator of quality for edible oils. Therefore, the quantitative analysis of fatty acids is often used for assessing oil quality and discrimination of edible oil adulteration. In this paper, four fatty acids (i.e., palmitic acid, stearic acid, arachidic acid, and behenic acid) were chosen as the objects of study. The reference values of four fatty acids in different edible oils were determined by GC-MS method. The accurate quantification of fatty acids in different edible oil samples were measured by the GCMS-QP2010 SE (Shimadzu Corporation, Japan) with the DB-5MS gas chromatographic column (30 m × 0.25 mm × 0.25 um). The purity 99.99% helium with the constant flow rate 1 mL/min was used as the carrier gas in the experiment. The fatty acid contents of the edible oils were analyzed after derivatization to their methyl ester products. The oil samples were preprocessed by four corresponding methyl esters (Methyl palmitate, Methyl stearate, Methyl arachidate, and Methyl behenate) in different concentrations to conduct the hot boiling separation because the boiling points of the derivatives from methyl esterification varied in a long range compared to the primitive forms. The detailed experimental process of methyl esterification was provided in Supplementary Materials, as shown in Figures S1 and S2 and Table S1. The oil samples were processed with methyl esterification and measured by the GC-MS to quantify the content of palmitic acid, stearic acid, arachidic acid, and behenic acid.

### 2.4. Pretreatment of Vis-NIR Reflectance Spectra

Although the precautions (the black box environment and the whiteboard calibration) had been taken in the spectral measurement system, the raw spectra are inevitable to suffer from the system noise and disturbance from measuring environment including light scattering, temperature, baseline migration and others. The multivariate scattering correction (MSC) [23], standard normal variate (SNV) [24], savitzky-golay (SG) smoothing [25], and wavelet transform (WT) [26] were applied for the Vis-NIR reflectance spectra to correct spectral data. The MSC and SNV are used to eliminate the effect of surface scattering and optical path variation in spectra data. The SG smoothing is an effective algorithm for improving spectral signal-to-noise ratio. The WT is commonly used for spectrum filtering and noise reduction. The four pretreatment algorithms were used to preprocess the reflectance spectra. Besides, the raw spectra without any pretreatments were also considered for model establishment to evaluate the effectiveness of different pretreatments methods in the application of Vis-NIR reflectance spectra.

### 2.5. Selection of Effective Wavelengths of Vis-NIR Reflectance Spectra

In addition, the obtained Vis-NIR reflectance spectra with full wavelengths were a high-dimensional data matrix. A large amount of redundant and irrelevant spectral information was mixed in the data. The effective wavelength selection was of important for establishing simplified and stabilized prediction models. In this paper, the successive projections algorithm (SPA) [27], variable importance of projection (VIP) [28] and principal component analysis (PCA) [29] were used for spectra feature extraction and wavelength selection.

- Successive projections algorithm (SPA): SPA is a variable-selection technique that selects variables with minimal redundant information and collinearity from the spectral information. It is a forward selection method by calculating the projection of each wavelength on the other unselected wavelengths and introducing the wavelengths with maximum projection into the combination of wavelengths.
- Variable importance in projection (VIP): VIP is an analytical technique for estimating the effect of individual variables in a system. The VIP score is a parameter used to evaluate the importance of the independent variable to the dependent variable in the model. An independent variable with a higher score is considered as significant influence on the dependent variable. Variables with low scores are discarded to ensure the validity of the model.
- Principal component analysis (PCA): PCA is a statistical analysis method that can reduce and simplify the original data. The spectra had a wide range of bands with a certain correlation between different bands. The generated principal components are the comprehensive indices by the linear combination of the primitive features (i.e., different wavelengths in this study), that can eliminate the correlation in original data. The loading vectors of PCA can be used to select the important wavelength regions. The higher the loading values, the more important the corresponding wavelengths. The wavelength points with the larger absolute values in the top loading vectors were selected as the effective wavelengths.

### 2.6. Models Establishment

The regression models for fatty acid contents prediction were developed by PLSR, SVM and RF. PLSR is a linear regression method to process high-dimensional regressors of one or several response variables [30]. A linear regression model is constructed based on a small number of latent variables which are the projection of explanatory variables and response variables in new space. The obtained latent variables have the maximum covariance between the new explanatory variables and the new response variables. PLSR possesses good performance in prediction analysis of spectrum data and has been widely used in chemometrics. The main idea of SVM is to convert the inputs from a low-dimensional feature space to a high-dimensional feature space [31]. The SVM applies a kernel function to construct an optimal hyperplane for separating the samples in high-dimensional feature space with good theoretical properties in generalization and convergence. Although the SVM is proposed for classification, it could also be used to regression analysis and has outstanding performance in spectral data analysis field [32]. RF is a non-linear ensemble method for prediction analysis. A series of simple decision trees are generated based on an injection of randomness strategy and all the prediction results are integrated as the final result. The result of classification or regression is the mean value of a large number of the generated decision trees [33,34].

### 2.7. Performance Evaluation

To assess the prediction performance of the three regression models SVM, RF, and PLSR, the obtained spectra of different edible oil samples were divided into calibration set and prediction set. The 70% spectra of a kind of oil were randomly split into calibration set and the rest were taken as the prediction set. The calibration set and the prediction set were used to train the model and evaluate model performance, respectively. The model performance was quantitatively evaluated using coefficients of determination of calibration set ($R_{C}^{2}$) and prediction set ($R_{P}^{2}$), and the root mean square errors of calibration set ($RMSE_{C}$) and prediction set ($RMSE_{P}$) as follow equations (Equations (2) and (3)):

(2) $$R^{2}=1-\frac{RSS}{TSS}$$

(3) $$RMSE=\sqrt{\frac{\sum_{i=1}^{N}{\left(y_{i}-y_{p}\right)}^{2}}{N}}$$

where $R^{2}$ is the coefficient of determination, $RSS=\sum_{i=1}^{N}{\left(y_{i}-y_{p}\right)}^{2}$ is the residual sum of squares and $TSS=\sum_{i=1}^{N}{\left(y_{i}-\bar{y}\right)}^{2}$ is total sum of squares. $y_{i}$ is the reference value of the experimental oil sample, $y_{p}$ is the predicted value for the oil sample, and $\bar{y}$ is mean of the reference values. *N* is the number of the oil samples.

The SVM, RF, and PLSR models were performed in MATLAB (Version: R2019b, Mathworks, Inc., Natick, MA, USA). The experiment computer used an Intel core i7-8700 CPU with a main frequency of 3.7 GHZ.

## 3. Results and Discussion

### 3.1. Vis-NIR Reflectance Spectra of Different Edible Oils

**Figure 2:**
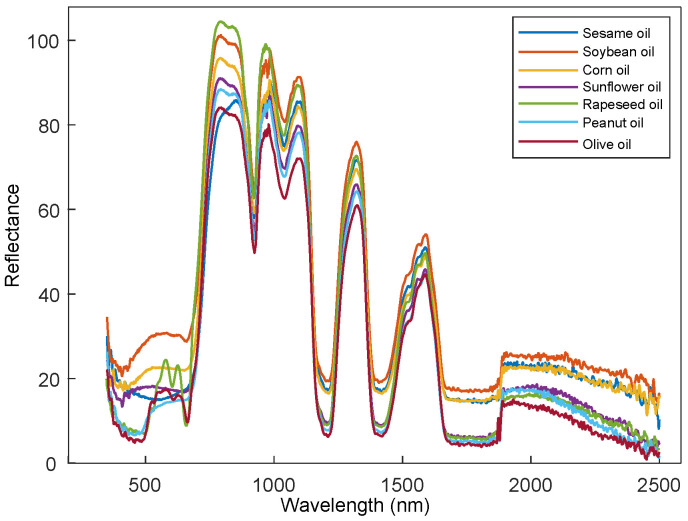
The representative Vis-NIR reflectance spectra of the seven kinds of edible oils.

The spectra of the 93 samples including seven kinds of edible oils were measured by the constructed hyperspectral spectroradiometer system. The representative spectra of the seven kinds of edible oils were shown in Figure 2. The spectral properties of the 7 kinds of edible oils can be classified into three categories. The spectra of soybean oil, corn oil, sunflower oil, and peanut oil showed the similar trends in visible region with a gentle absorption peak (around 510 nm). The spectra of rapeseed oil and olive oil showed the multiple absorption peaks (585 nm and 631 nm) in visible region, which were markedly different with the above four edible oils. The spectra of sesame oil had no absorption peak in visible region which was unlike all other edible oils. On the whole, the spectra of the seven kinds of edible oils in NIR range (780–2500 nm) performed very similar overall trends. However, the spectra of different edible oils vary in spectral reflectance and spectral shape in some specific narrow regions. The spectral peak at wavelength 800 nm is related to O-H first stretching overtone. The spectral peaks at wavelengths 856 nm and 1098 nm belong to the C-H third overtone [35], and the spectral peak in wavelength 1586 nm is for the second overtone of N-H [36]. The peak at around wavelength 1320 nm is related to C-H combinations, and the peak at wavelength 980 nm is related to the second overtone of O-H bending [37,38].

### 3.2. Quantitative Determination of Fatty Acids by GC-MS

The reference values of the fatty acids in edible oils need to be accurately obtained for model establishment and model validation. In this study, the oil sample of each brand was measured by GC-MS. All the oil samples were methylated by four FAMEs. The complete data of the content of the four fatty acids measured by GC-MS was provided in Supplementary Materials (Table S2). The quantitative results of all 93 brands in seven kinds of oils were analyzed as follows.

**Figure 3:**
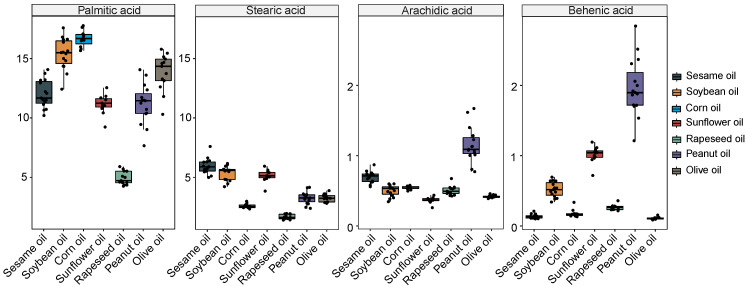
The box plots of the contents of four fatty acids in different edible oils (Unit: %). The horizontal black line in each bar box was the median value of the fatty acids in each kind of oils. The top and bottom lines of each box were the positions of 75% and 25% of the values, respectively. The black dots represented the oil samples. Some of the dots which exceeded the top or bottom of the vertical line of the bar box were considered as outliers from the overall level.

First, the statistical analysis of quantitative results of four fatty acids were shown in Table 1 and Figure 3. For the palmitic acid, except rapeseed oil, the other six kinds of edible oils were detected with high contents. The corn oil was rich in palmitic acid (mean = 16.67%, standard deviation (sd) = 0.68) compared to other oils. The rapeseed oil had the lowest content of palmitic acid (mean = 4.93%, sd = 0.57). For the stearic acid, the contents in all oil samples were less than the palmitic acid. The average content of stearic acid in seven kinds of edible oils ranged from 1.71% to 5.97%. The standard deviations of the stearic acid content in seven kinds of edible oils were less than 0.65, which represented that the stearic acid content varied very small in all kinds of the oils. For the arachidic acid and behenic acid, the contents were relatively rare in all the seven kinds of oils. Comparatively, the peanut oil was more rich in the arachidic acid and behenic acid than other oils. The variation levels of arachidic acid and behenic acid in peanut oil were relatively larger than other edible oils (as shown in Figure 3). The standard deviations of arachidic acid and behenic acid in peanut oil were 0.25 and 0.41, respectively. Moreover, the results of Wilcoxon test of four fatty acids between the pairs of the edible oils were shown in the Supplementary Materials (Figure S3).

**Figure 4:**
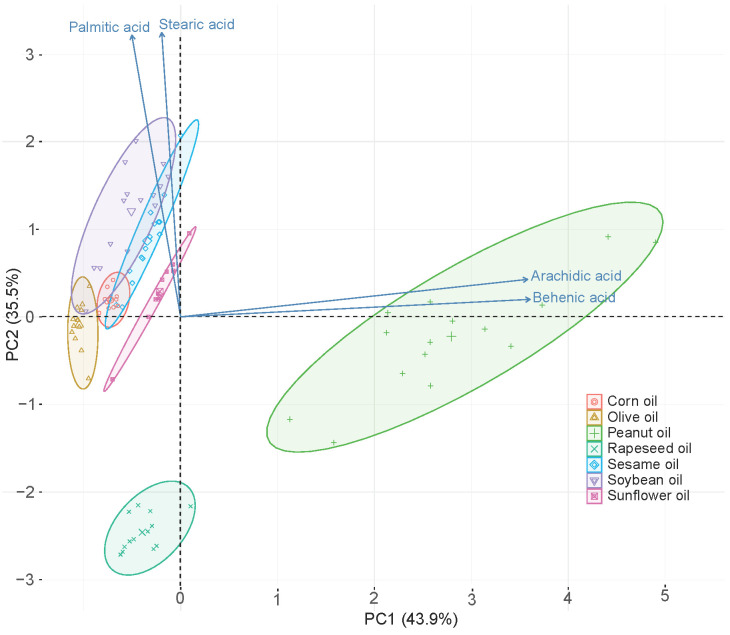
The visualization of the quantitative results of four fatty acids in seven kinds of edible oils.

In addition, the quantitative data of the four fatty acids were analyzed by PCA to give an intuitive visualization of the distribution of 93 oil samples. In the PCA analysis, the quantitative data of the four fatty acids in 93 oil samples were normalized with mean 0 and variance 1. The top two principal components were extracted for visualizing the distribution of different oil samples, as shown in Figure 4. Three kinds of edible oils (rapeseed oil, peanut oil, and sunflower oil) were in their own completely independent population. The 13 brands of rapeseed oils had a high consistency in compact category. While different brands of peanut oils showed a degree of dispersion, they had distinct distinguishing features with other oils. The sunflower oils also showed a clear independent category. Three kinds of oils (corn oil, olive oil, and sesame oil) were located on their own separate groups. However, the dispersion of 15 brands of soybean oils was relatively large and crosslinked with three oil groups (corn oil, olive oil and sesame oil). Overall, each kind of oil shows distinct categorical features. This analysis demonstrated that the quantitative analysis of the four fatty acids is effective to distinguish different kinds of edible oils.

### 3.3. Prediction of Fatty Acid Contents with Full Wavelengths Reflectance Spectra

**Figure 5:**
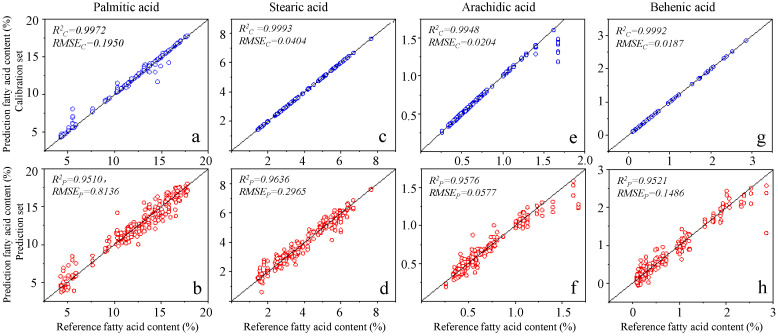
The results of palmitic acid on calibration set (**a**) and prediction set (**b**) using full wavelengths in the optimal regression model; the prediction results of stearic acid on calibration set (**c**) and prediction set (**d**) using full wavelengths in the optimal regression model; the prediction results of arachidic acid on calibration set (**e**) and prediction set (**f**) using full wavelengths in the optimal regression model; the prediction results of behenic acid on calibration set (**g**) and prediction set (**h**) using full wavelengths in the optimal regression model. ($R_{C}^{2}$: coefficient of determination in calibration set; $R_{P}^{2}$: coefficient of determination in prediction set; $RMSE_{C}$: root mean square error in calibration set; $RMSE_{P}$: root mean square error in prediction set).

The PLSR, SVM, and RF coupled with multiple pretreatments of oil spectra were used to develop the prediction models for the quantitative analysis of the content of fatty acids in different edible oils. Each of the regression methods was combined with the four pretreatment algorithms (SNV, MSC, SG smoothing, and WT) and the raw spectra to train the models in calibration set. The top two pretreatment methods yielded the best performance in each regression model were shown in Table 2, and the parameter setting of the multivariate analysis models were provided in Table S4. As seen from Table 2, as a whole, the performance of SVM model for the four fatty acids were better than PLSR and RF model. Therefore, the SVM regression model was suitable for the quantitative analysis of the content of fatty acids. For palmitic acid, the best prediction result was obtained by SVM model constructed with spectral data preprocessed by MSC pretreatment, which had $R_{C}^{2}$ of 0.9972, $RMSE_{C}$ of 0.1950, $R_{P}^{2}$ of 0.9510, and $RMSE_{P}$ of 0.8136. The corresponding scatter plots of prediction performance on the calibration set and prediction set were shown in Figure 5a,b, respectively. However, the second place, the model of SVM with SNV pretreatment had the very similar performance as the model of SVM with MSC pretreatment. For the stearic acid, the regression model constructed by SVM model combined with SNV pretreatment had the best prediction effect with $R_{C}^{2}$ and $RMSE_{C}$ being 0.9993 and 0.0404 on the calibration set, $R_{P}^{2}$ and $RMSE_{P}$ being 0.9636 and 0.2965 on the prediction set, respectively. The prediction performance was demonstrated in Figure 5c,d. For the arachidic acid, the highest prediction accuracy was obtained using SVM model with SNV pretreatment which had $R_{C}^{2}$ = 0.9948, $RMSE_{C}$ = 0.0204 (as shown in Figure 5e), $R_{P}^{2}$ = 0.9576, $RMSE_{P}$ = 0.0577 (as shown in Figure 5f). In addition, the model of SVM with SNV pretreatment for the spectral data provided the optimal prediction result in behenic acid group as evidenced by $R_{C}^{2}$ = 0.9992, $RMSE_{C}$ = 0.0187 (as shown in Figure 5g), $R_{P}^{2}$ = 0.9521, $RMSE_{P}$ = 0.1486 (as shown in Figure 5h). The complete prediction results of all the pretreatment methods are provided in the Supplementary Materials (Table S3). Overall, the performance of the regression models constructed by SNV pretreatment were generally better than those with MSC, WT, and SG smoothing pretreatment, and the raw spectra. Therefore, the SNV pretreatment was adopted as the standard method for preprocessing the original spectral data in the subsequent analysis. It was worth noting that the constructed PLSR models were with the large number of latent variables (LVs). In our experiments, we tested different numbers of latent variables (LVs) in PLSR and chose the model with the best prediction performance. However, the large number of LVs could lead to overfitting of PLSR models. It should be very careful to choose the appropriate number of LVs in PLSR in different studies. The prediction results for the four fatty acids led by Vis-NIR reflectance spectroscopy were in a good agreement with the quantitative results obtained by GS-MS but with a very significant rapid detection in a measuring time. Thus, this study presented an effective approach for rapid detection of fatty acids of edible oils. The four fatty acid contents in edible oils were accurately quantified using the multivariate data processing and analysis methods on Vis-NIR reflectance spectroscopy.

### 3.4. Prediction of Fatty Acid Contents with Effective Wavelengths

**Figure 6:**
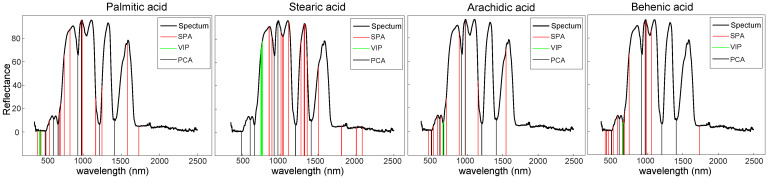
Effective wavelengths selected by SPA, VIP, and PCA for quantitative analysis of four fatty acids. The red vertical line corresponded to the effective wavelengths selected by SPA, the green vertical line corresponded to the effective wavelengths selected by VIP, and the black vertical line corresponded to the effective wavelengths selected by PCA. The spectrum of rapeseed oil was chosen as the background reference. (SPA: successive projections algorithm, VIP: variable importance in projection, PCA: principal component analysis).

A substantial proportion of redundant and irrelevant information was comprised in the high dimension of Vis-NIR reflectance spectra data. Extracting effective wavelengths could stabilize the prediction model and improve the computational efficiency. In order to eliminate the redundant information in the spectra and simplify the model to develop the real-time detection instrument for the prediction of fatty acid contents in edible oils, the SPA, VIP, and PCA algorithms were used to extract the main information and reduce the dimension of the spectra of edible oils. Based on the analytical results of the full wavelengths, the SNV pretreatment was applied for preprocessing of spectra data. The processed spectra were used to develop the regression models using RF, SVM, and PLSR. The best models with SPA, VIP, and PCA algorithms in each of the fatty acids were shown in Table 3, and the parameter setting of multivariate analysis models were provided in Table S5. The effective wavelengths selected by the SPA, VIP, and PCA were shown in Figure 6. The VIP algorithm only picked out few continuous wavelengths in visible band in all the four fatty acid experiments with very poor prediction results (as shown in Table 3), which demonstrated that the visible wavelengths were not enough to distinguish the components in edible oils. The PCA inclined to select the wavelengths in peaks and troughs of the spectra. Similarly, the effective wavelengths selected by PCA algorithm did not help to generate the good prediction results in all the four fatty acids (as shown in Table 3). It indicated that the peaks and troughs of the spectra were not good indicators for discrimination of components in edible oil based on Vis-NIR reflectance spectroscopy. In contrast, the SPA algorithm was capable to select the finite number of discrete wavelengths including visible and NIR bands as the effective wavelengths. Comparing with the effective wavelengths selected by VIP and PCA, the regression models constructed with the effective wavelengths selected by SPA yielded a significant improvement of prediction effects on the content of all four fatty acids in edible oils.

**Figure 7:**
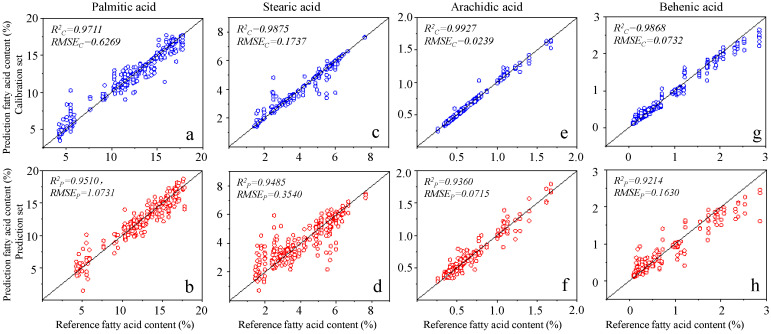
The results of palmitic acid on calibration set (**a**) and prediction set (**b**) using the selected wavelengths in the optimal regression model; the results of stearic acid on calibration set (**c**) and prediction set (**d**) using the selected wavelengths in the optimal regression model; the results of arachidic acid on calibration set (**e**) and prediction set (**f**) using the selected wavelengths in the optimal regression model; the results of behenic acid on calibration set (**g**) and prediction set (**h**) using the selected wavelengths in the optimal regression model. ($R_{C}^{2}$: coefficient of determination in calibration set; $R_{P}^{2}$: coefficient of determination in prediction set; $RMSE_{C}$: root mean square error in calibration set; $RMSE_{P}$: root mean square error in prediction set).

To be more specific, for the palmitic acid, the SPA decreased the number of wavelengths from 2151 to 17 ( 819, 744, 973, 1159, 698, 664, 968, 1729, 680, 549, 503, 1576, 1242, 426, 980, 970, and 392 nm). The best prediction results were $R_{C}^{2}$ = 0.9711, $RMSE_{C}$ = 0.6269, $R_{P}^{2}$ = 0.915, and $RMSE_{P}$ = 1.0731. The prediction results were shown in Figure 7a,b. In the prediction of stearic acid content, the discrete wavelengths (1317, 1811, 855, 2082, 1276, 1505, 762, 1012, 893, 1323, 1029, 2005, 1111, 1356, and 1044 nm) were screened by SPA which achieved the best prediction accuracy with $R_{C}^{2}$ = 0.9875, $RMSE_{C}$ = 0.1737, $R_{P}^{2}$ = 0.9485, and $RMSE_{P}$ = 0.3540. The corresponding prediction results were shown in Figure 7c,d. For arachidic acid, the 11 wavelengths (499, 626, 455, 887, 522, 579, 1546, 973, 1152, 974, and 705 nm) were screened by SPA with the model performance as $R_{C}^{2}$ = 0.9927, $RMSE_{C}$ = 0.0239, $R_{P}^{2}$ = 0.9360, $RMSE_{P}$ = 0.0715 and the prediction effects were shown in Figure 7e,f. In the quantitative analysis of behenic acid content, the 14 wavelengths selected by SPA were 649, 455, 498, 1058, 519, 681, 985, 1739, 426, 666, 406, 578, 974, and 744 nm. The constructed RF regression model had the best prediction effect with $R_{C}^{2}$ and $RMSE_{C}$ were 0.9868 and 0.0732, respectively. The $R_{P}^{2}$ and $RMSE_{P}$ were 0.9214 and 0.1630, respectively. The prediction results were shown in Figure 7g,h. In summary, the prediction results of the simplified regression model constructed by the effective wavelengths proved that the characteristic wavelength selection method SPA was effective to construct a useful model for the rapid quantitative analysis of four fatty acids in edible oils. This result is in accordance with the previous research findings [39]. In addition, the simplified regression models can also be used to design a fast and portable Vis-NIR reflectance spectroscopy system to detect the fatty acid contents in edible oils.

## 4. Conclusions

In this study, an efficient and non-destructive method using Vis-NIR spectroscopy was presented for fatty acids identification of edible oils. The GC-MS was used to determine the content of palmitic acid, stearic acid, arachidic acid and behenic acid in 93 brands of edible oils as the reference values. The Vis-NIR spectroscopy of the 93 oil samples in different brands were measured by a constructed hyperspectral spectroradiometer system for model establishment and prediction. Overall, the prediction results showed that the SVM regression model with SNV pretreatment on full wavelengths had the best predictive effect on the four fatty acids. For the prediction of palmitic acid, the model of SVM with SNV pretreatment had $R_{P}^{2}$ = 0.9504 and $RMSE_{P}$ = 0.8181, the best model for stearic acid was SVM with SNV pretreatment which had $R_{P}^{2}$ = 0.9636 and $RMSE_{P}$ = 0.2965, the best model for arachidic acid was SVM with SNV pretreatment which had $R_{P}^{2}$ = 0.9576 and $RMSE_{P}$ = 0.0577, and the best model for behenic acid was SVM with SNV pretreatment which had $R_{P}^{2}$ = 0.9521 and $RMSE_{P}$ = 0.1486. In addition, three algorithms SPA, VIP, and PCA were evaluated systematically for the effectiveness of constructing a simplified but stabilized model by selecting the effective wavelengths. The VIP algorithm was only capable to find the continuous wavelengths in visible region. The PCA algorithm was also not an ideal algorithm for effective wavelengths screening in the Vis-NIR spectra of edible oils. In contrast, the SPA algorithm extracted the effective wavelengths distributed in visible and NIR bands. The regression model constructed with the effective wavelengths screened by SPA had ideal performance in the prediction of all the four fatty acids. Most of the results based on the effective wavelengths expressed a certain degree of degradation comparing with the results from full wavelengths. Even so, the loss of prediction accuracy was small and tolerable. Accordingly, the Vis-NIR spectroscopy with multivariate methods led to the accurate and rapid detection of fatty acid contents in edible oils. The simplified regression models constructed by the effective wavelengths are benefit for facilitating more faster and convenient analysis in the fatty acids detection of edible oils. However, the potential cause of the visible spectrum contributing to the prediction of fatty acid contents was not discussed in this paper and should be further studied in future.

## Figures and Tables

**Table 1:** The statistics of the four fatty acids in the seven kinds of edible oils (units: %).

Oil	Palmitic Acid			Stearic Acid			Arachidic Acid			Behenic Acid		
	Range	Mean	sd	Range	Mean	sd	Range	Mean	sd	Range	Mean	sd
Sesame oil	14.07–10.2	12.01	1.18	7.64–5.03	5.97	0.65	0.87–0.7	0.7	0.08	0.22–0.11	0.14	0.03
Soybean oil	17.58–12.42	15.38	1.34	6.21–4.26	5.4	0.6	0.6–0.5	0.5	0.08	0.7–0.35	0.53	0.11
Corn oil	17.76–15.66	16.67	0.68	3.02–2.38	2.63	0.19	0.58–0.54	0.54	0.02	0.34–0.14	0.19	0.06
Sunflower oil	12.54–9.23	11.17	0.86	5.98–3.88	5.16	0.54	0.44–0.37	0.37	0.04	1.2–0.73	1.02	0.12
Rapeseed oil	5.9–4.26	4.93	0.57	2–1.44	1.71	0.2	0.67–0.51	0.51	0.07	0.37–0.22	0.27	0.04
Peanut oil	14.06–7.66	11.18	1.69	4.2–2.43	3.31	0.52	1.67–1.16	1.16	0.25	2.85–1.22	1.98	0.41
Olive oil	15.78–10.3	13.83	1.58	3.92–2.86	3.3	0.33	0.46–0.42	0.42	0.02	0.16–0.09	0.12	0.02

Range: the maximum and minimum content in the oils. sd: standard deviation.

**Table 2:** Prediction results of four fatty acids in edible oils using full wavelengths.

Fatty Acids	Model	Pretreatment	Calibration Set		Prediction Set	
			$R_{C}^{2}$	${\mathrm{RMSE}}_{C}$	$R_{P}^{2}$	${\mathrm{RMSE}}_{P}$
Palmitic acid	PLSR	SNV	0.9402	0.7583	0.8807	1.5326
		MSC	0.9403	0.7579	0.8733	1.6297
	SVM	SNV	0.9952	0.2562	0.9504	0.8181
		MSC	0.9972	0.195	0.951	0.8136
	RF	SNV	0.9833	0.4215	0.8552	1.0355
		MSC	0.9828	0.4288	0.8572	1.0418
Stearic acid	PLSR	SNV	0.9257	0.4233	0.8607	0.5845
		MSC	0.9258	0.4232	0.8553	0.5968
	SVM	SNV	0.9993	0.0404	0.9636	0.2965
		MSC	0.9956	0.1035	0.9624	0.3016
	RF	SNV	0.9857	0.1735	0.9126	0.3954
		MSC	0.9866	0.1676	0.9168	0.3847
Arachidic acid	PLSR	SNV	0.9008	0.0879	0.8186	0.1203
		WT	0.8838	0.0952	0.8174	0.1211
	SVM	SNV	0.9948	0.0204	0.9576	0.0577
		MSC	0.9907	0.0276	0.9526	0.0615
	RF	SNV	0.9839	0.0317	0.9414	0.0548
		MSC	0.9843	0.0317	0.9421	0.0562
Behenic acid	PLSR	SNV	0.9324	0.176	0.8699	0.2485
		SG smoothing	0.9	0.214	0.8701	0.2459
	SVM	SNV	0.9992	0.0187	0.9521	0.1486
		MSC	0.9993	0.0184	0.9485	0.1543
	RF	SNV	0.9905	0.0622	0.9486	0.1359
		MSC	0.9915	0.0589	0.9496	0.1347

$R_{C}^{2}$: coefficient of determination in calibration set; $RMSE_{C}$: root mean square error in calibration set; $R_{P}^{2}$: coefficient of determination in prediction set; $RMSE_{P}$: root mean square error in prediction set; PLSR: partial least squares regression; RF: random forest; SVM: support vector machine; SNV: standard normal variables; MSC: multivariate scattering correction; WT: wavelet transform.

**Table 3:** Prediction results of four fatty acids in edible oils by the better model using effective wavelengths of spectra.

Fatty Acid	Model	Selected Wavelength (nm)	Calibration Set		Prediction Set	
			$R_{C}^{2}$	${\mathrm{RMSE}}_{C}$	$R_{P}^{2}$	${\mathrm{RMSE}}_{P}$
Palmitic acid	SNV + SPA + SVM	819 744 973 1159 698 664 968 1729 680 549 503 1576 1242 426 980 970 392	0.9711	0.6269	0.915	1.0731
	SNV + VIP + SVM	437 418 439 416 417 438	0.4632	2.7038	0.3809	2.8959
	SNV + PCA + SVM	493 605 663 922 971 1205 1409	0.8393	1.481	0.7872	1.7138
Stearic acid	SNV + SPA + SVM	1317 1811 855 2082 1276 1505 762 1012 893 1323 1029 2005 1111 1356 1044	0.9875	0.1737	0.9485	0.354
	SNV + VIP + SVM	762 747 755 765 767 766 757 752 756 753 754 751 748 750	0.7987	0.6972	0.6089	0.9933
	SNV + PCA + SVM	493 605 663 922 971 1205 1409	0.735	0.8044	0.6306	0.9461
Arachidic acid	SNV + SPA + SVM	499 626 455 887 522 579 1546 973 1152 974 705	0.9927	0.0239	0.936	0.0715
	SNV + VIP + SVM	670 656 669 657 668 658 667 666 659 665 660 664 661 662 663	0.3978	0.2192	0.3177	0.2333
	SNV + PCA + RF	493 605 663 922 971 1205 1409	0.9689	0.0424	0.8377	0.0849
Behenic acid	SNV + SPA + RF	649 455 498 1058 519 681 985 1739 426 666 406 578 974 744	0.9866	0.0735	0.9229	0.1606
	SNV + VIP + SVM	669 655 668 656 667 657 666 665 658 664 659 663 662 660 661	0.4743	0.4977	0.4561	0.5097
	SNV + PCA + RF	493 605 663 922 971 1205 1409	0.9716	0.1014	0.8462	0.2121

$R_{C}^{2}$: coefficient of determination in calibration set; $RMSE_{C}$: root mean square error in calibration set; $R_{P}^{2}$: coefficient of determination in prediction set; $RMSE_{P}$: root mean square error in prediction set; SNV: Standard normal variables; VIP: variable importance in projection; PCA: principal component analysis; SVM: support vector machine; RF: random forest.

## Data Availability

Not applicable.

## Supplementary Materials

The following are available at https://www.mdpi.com/article/10.3390/bios11080261/s1, Figure S1: The workflow of the quantitative analysis for fatty acids in oil samples by GC-MS, Figure S2: The representative chromatograms of four fatty acids composition of sesame oil, Figure S3: The statistical tests of four fatty acids in different edible oils, Table S1: Qualitative analysis of four FAMEs by GC-MS, Table S2: The quantitative results of four fatty acids in 93 brands of edible oils by GC-MS, Table S3: Prediction results of four fatty acids in edible oils obtained using full wavelengths, Table S4: Parameter setting of multivariate analysis methods using the full wavelengths, Table S5: Parameter setting of multivariate analysis methods using the effective wavelengths.
