# Supplementary material for: Rapid Detection of Fatty Acids in Edible Oils Using Vis-NIR Reflectance Spectroscopy with Multivariate Methods
Source: Biosensors (Basel). 2021 Aug 3;11(8):261. doi: 10.3390/bios11080261 (PMC8395004; doi:10.3390/bios11080261)
Supplement: Supplementary file 1 [file biosensors-11-00261-s001.zip › biosensors-1287488-supplementary.pdf]

Supplementary material: Rapid quality detection of edible oils using Vis-NIR reflectance spectroscopy with multivariate methods

Ning Su [1,2], Fangfang Pan [3], Liusan Wang [1,2,*], and Shizhuang Weng[3,*]

[1] Institute of Intelligent Machines, Hefei Institutes of Physical Science, Chinese Academy of Sciences, Hefei 230031, China
[2] Intelligent Agriculture Engineering Laboratory of Anhui Province, China, Hefei 230031, China
[3] National Engineering Research Center for Agro-Ecological Big Data Analysis and Application, Anhui University, Hefei 230601, China
[*] Correspondence:lswang@iim.ac.cn (L. W.), weng_1989@126.com (S. W.)

Measurement of fatty acid contents by GC-MS

The workflow of the quantitative determination for fatty acids in oil samples by GC-MS is shown in Fig. S1. First, the 100 mg of oil sample was dissolved in 2 mL of n-hexane and oscillated by ultrasonic waves for 10 minutes. Then, the solution was added 2 mL of methanol solution (0.5 mol /L) and ultrasonic vibration for 5 minutes to methyl esterification of the oil sample. Third, 2ml hydrochloric acid was added and settled in a warm bath for 3 minutes to neutralize excess potassium hydroxide. At last, the final organic solution at the top level was separated by 0.4 μm filter. The processed sample was measured by GCMS-QP2010 SE.

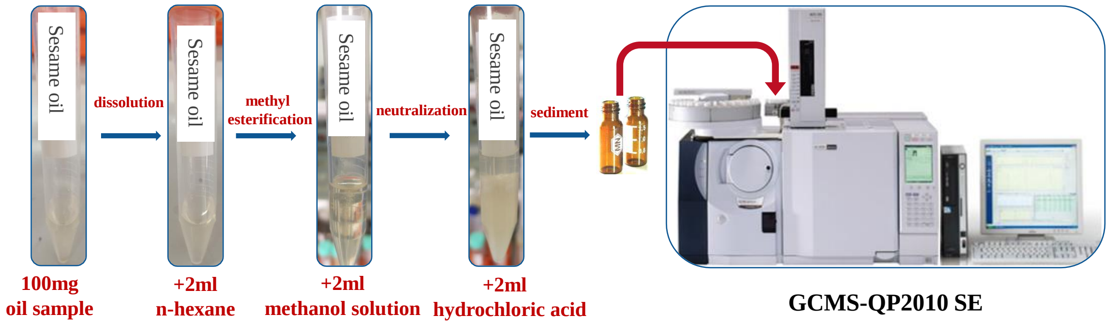

Figure S1. The workflow of the quantitative analysis for fatty acids in oil samples by GC-MS.

Five standard samples of fatty acid methyl esters with different concentrations were used to observe the content of four FAMEs. The GC-MS was used to collect the ion spectra of the four FAMEs mixed standard samples. The deterministic quantitative relationship of the four fatty acids were shown in Table S1.

Table S1. Qualitative analysis of four FAMEs by GC-MS

Composition	Structure	Time (min)	Target (m/z)	Calibration curve	R^2
Methyl palmitate	C16:0	12.975	270.45	$y = 1447802x$	0.9835
Methyl stearate	C18:0	14.683	298.50	$y = 1915373x$	0.9999
Methyl arachidate	C20:0	16.833	326.56	$y = 1818904x$	0.9989
Methyl behenate	C22:0	19.558	354.61	$y = 1818514x$	0.9999

x is the percentage of fatty acid content and y is the peak area, R^2 is the coefficient of determination of the model.

Based on the measurement of GC-MS, the representative chromatograms of the four fatty acids in seven kinds of edible oils were shown in Fig. S2. The chromatographic peak 1 corresponds to methyl palmitate. The chromatographic peak 2 corresponds to methyl stearate. The chromatographic peak 3 corresponds to methyl arachidate. The chromatographic peak 4 corresponds to methyl behenate. The complete data of the content of the four fatty acids was provided in Table S2.

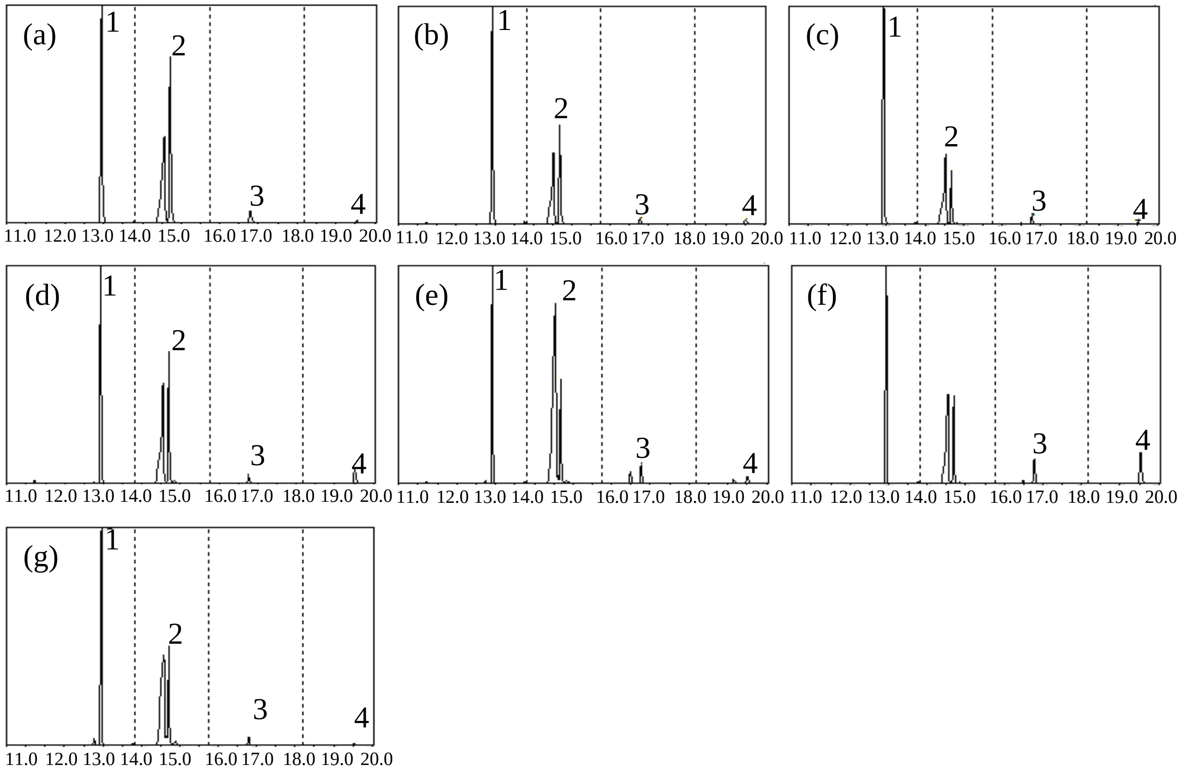

Figure S2. The representative chromatograms of four fatty acids composition of sesame oil (a), soybean oil (b), corn oil (c), sunflower oil (d), rapeseed oil (e), peanut oil (f), and olive oil (g).

Table S2. The quantitative results of four fatty acids in 93 brands of edible oils by GC-MS (Unit:%)

Label	Palmitic acid	Stearic acid	Arachidic acid	Behenic acid
Three Tim Sesame Oil	11.133	6.4327	0.7114	0.122
Arowana Sesame Oil	10.2031	5.1494	0.5798	0.1104
Lee Kum Kee Pure Black Sesame Oil	10.7171	5.5025	0.6271	0.1174
Totole Sesame Oil	11.6748	5.7126	0.6716	0.1309
Longxi Sesame Oil	12.207	6.3237	0.7537	0.1421
Sharia Sesame Oil	10.6582	5.0288	0.559	0.108
Yanzhuang Sesame Oil	11.5204	5.9869	0.6898	0.1304
Luhua Sesame Oil	11.3329	5.5537	0.6228	0.1183
Jiudouwan Sesame Oil	13.2082	5.5894	0.7411	0.1815
Xiangmanyuan Sesame Oil	13.1675	5.9234	0.7405	0.1774
Changkang Sesame Oil	11.4941	5.7723	0.639	0.2166
Cuiyu Sesame Oil	12.9455	6.674	0.7818	0.149
Lee Kum Kee Pure Sesame Oil	12.0693	6.3515	0.7316	0.141
Arowana small grinding Sesame Oil	13.7509	5.9319	0.7234	0.1549
Xingzhenhu Sesame Oil	14.0677	7.6389	0.8711	0.1466
Ginger Soybean Oil	12.4161	4.2558	0.3455	0.347
Guiqingyuan Soybean Oil	13.7001	4.7536	0.4013	0.3988
Arowana Soybean Oil	14.34	4.8426	0.4702	0.5239
Laki Soybean Oil	14.3579	4.4982	0.385	0.3902
Shuheyou Soybean Oil	14.813	4.8688	0.4321	0.4598
Fuhong Soybean Oil	15.0004	5.6376	0.5685	0.5986
Fulinmen Soybean Oil	15.4023	5.6399	0.5348	0.5466
Jiusan Soybean Oil	15.4915	5.631	0.4899	0.4888
Qishi Soybean Oil	15.6377	5.7207	0.5023	0.4984
Yupinxiang Soybean Oil	15.5263	5.686	0.5467	0.6475
Shukeman Soybean Oil	16.6229	5.9816	0.6	0.6549
Tianxiawugu Soybean Oil	16.3757	5.5266	0.5834	0.6472
Zhongding Soybean Oil	16.5944	6.1095	0.5365	0.4295
Yuanbao Soybean Oil	16.7999	5.5772	0.5982	0.7016
Jilang Soybean Oil	17.5792	6.2082	0.5434	0.5475
West King Corn Oil	16.979	2.4463	0.5384	0.1667
Galaxy Corn Oil	16.4881	2.5038	0.5356	0.18
Jinding Corn Oil	16.5418	2.3833	0.5129	0.1387
Daomai Corn Oil	15.979	2.7365	0.5318	0.2292
Haitian Corn Oil	15.7946	3.0245	0.4969	0.343
Meiling Corn Oil	17.6201	2.559	0.5562	0.1478
Fengyuan Corn Oil	16.8488	2.5668	0.5489	0.1771
Kuiwang Corn Oil	15.6626	2.8855	0.5676	0.1817
Arowana Corn Oil	17.7624	2.6574	0.5712	0.1747
Jialebao Corn Oil	16.6837	2.6869	0.5779	0.1678
Fulinmen Corn Oil	17.0643	2.491	0.5396	0.1481
Chucui Sunflower Oil	11.2395	4.9457	0.3672	0.9748
Duoli Sunflower Oil	11.8228	5.1791	0.3674	1.0596
Fulinmen Sunflower Oil	11.0482	5.0737	0.3517	1.0517
Pietro Coricelli Sunflower Oil	11.5254	5.4915	0.3912	1.0479
Lizzi Sunflower Oil	9.2309	3.883	0.2605	0.7252
Arowana Sunflower Oil	11.6866	5.4341	0.3899	1.1208
Abril Sunflower Oil	10.7979	5.1255	0.37	0.9907

Haishi Sunflower Oil	10.4238	4.8667	0.3392	0.9504
Jinding Sunflower Oil	11.0386	5.1692	0.3654	1.0056
Haitian Sunflower Oil	11.5484	5.6514	0.4002	1.0905
Daomai Sunflower Oil	12.5424	5.9795	0.4374	1.2001
Tianfu Rapeseed Oil	4.5982	1.6598	0.4627	0.238
Xiancan Rapeseed Oil	4.3481	1.5791	0.5527	0.2813
Daodaoquan Rapeseed Oil	4.9927	1.6898	0.5287	0.2711
Linxiangyuan Rapeseed Oil	5.7023	1.9365	0.4648	0.2616
Fulinmen Rapeseed Oil	4.2581	1.5486	0.5343	0.2921
Haitian low erucic acid Rapeseed Oil	5.5169	1.9728	0.5405	0.2872
Fengyuan Rapeseed Oil	4.5607	1.5541	0.4389	0.2415
Nissin Rapeseed Oil	4.7172	1.651	0.4804	0.2394
Chuancaiwang Rapeseed Oil	4.5418	1.4439	0.4314	0.2373
Haitian Rapeseed Oil	4.3452	1.4594	0.4267	0.2224
Luhua Rapeseed Oil	5.9008	1.9958	0.494	0.2888
Arowana Rapeseed Oil	5.5283	1.9798	0.6746	0.3657
Hongqingting Rapeseed Oil	5.0887	1.7766	0.5462	0.2747
Hujihua Peanut Oil	11.4433	3.2881	1.2854	2.0055
Chucui Peanut Oil	13.5995	4.168	1.6716	2.852
Longda Puree Pressed Peanut Oil	14.0558	4.1993	1.6173	2.5231
Fulinmen Peanut Oil	10.3489	3.3056	1.2281	2.3765
Longda Squeezed Peanut Oil	10.4006	2.8187	1.0466	1.7244
Changshenghua Peanut Oil	10.72	3.1214	1.0786	1.8864
Jinsheng Peanut Oil	9.4494	2.8623	1.078	1.9047
First Place Peanut Oil	7.6572	2.4307	0.8053	1.5411
Chubao Peanut Oil	12.3571	3.5563	1.0129	1.7283
Xinghe Peanut Oil	12.7657	3.5506	1.1317	1.8719
Luhua Peanut Oil	11.7193	3.6458	1.4001	2.3164
Yuhuang Peanut Oil	11.2435	3.1966	1.0895	1.9271
Golden Valley Farm Peanut Oil	9.0187	2.5165	0.7728	1.2197
S1 Peanut Oil	11.4755	3.429	1.0037	1.7095
S2 Peanut Oil	11.4826	3.58	1.1298	2.0657
Luhua Olive Oil	14.588	3.9202	0.4582	0.1614
LaEspanola Olive Oil	10.3014	3.4154	0.4234	0.1121
Gallo Olive Oil	13.2771	3.6483	0.4169	0.1056
Big Ben Olive Oil	14.3439	3.2335	0.4054	0.1024
Olivier Olive Oil	15.7767	2.9086	0.4255	0.1234
Chudao Olive Oil	13.7346	3.2852	0.4141	0.1274
Albury Olive Oil	11.8003	3.5065	0.4122	0.1092
Muxi Manor Olive Oil	14.6688	2.922	0.4067	0.1071
Philippe Barry Olive Oil	14.9531	3.3215	0.4455	0.115
Bellina Olive Oil	15.4441	2.968	0.4407	0.121
Abaco Olive Oil	15.1407	2.8574	0.4228	0.1153
X1 Olive Oil	12.6099	3.6437	0.3899	0.0926
X2 Olive Oil	13.1325	3.2539	0.4005	0.1049

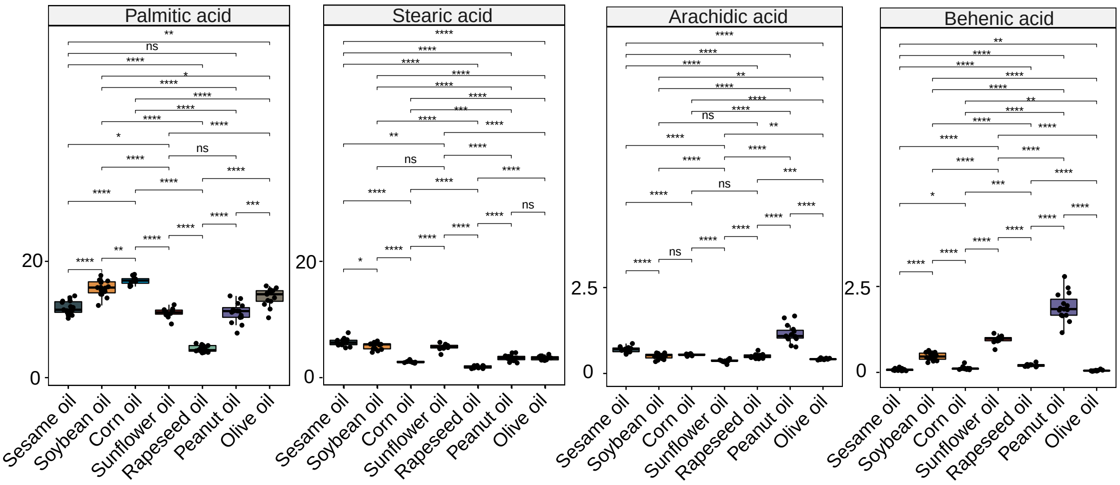

Figure S3. The statistical tests of four fatty acids in different edible oils.(**ns** represents P>0.05, * represents p <= 0.05, ** represents p <= 0.01, *** represents p <= 0.001, **** represents p <= 0.0001. The Wilcoxon test was used for the statistical test.)

Table S3. Prediction results of four fatty acids in edible oils obtained using full wavelengths

	Model	Pretreatment	Calibration set		Prediction set	
			R_C^2	$RMSE_C$	R_P^2	$RMSE_P$
Palmitic acid	PLSR	RAW	0.8365	0.9336	1.6649	0.8709
		SNV	0.7583	0.9402	1.5326	0.8807
		MSC	0.7579	0.9403	1.6297	0.8733
		SG smoothing	1.0946	0.9109	1.7507	0.8592
		WT	0.8849	0.9294	1.6768	0.8695
	SVM	RAW	0.6989	0.9645	1.1008	0.9117
		SNV	0.2562	0.9952	0.8181	0.9504
		MSC	0.195	0.9972	0.8136	0.951
		SG smoothing	0.7567	0.9582	1.12	0.9085
		WT	0.7105	0.9632	1.104	0.9111
	RF	RAW	0.5979	0.9662	1.3525	0.7893
		SNV	0.4215	0.9833	1.0355	0.8552
		MSC	0.4288	0.9828	1.0418	0.8572
		SG smoothing	0.599	0.9664	1.3786	0.7836
		WT	0.6096	0.9647	1.3762	0.7855
Stearic acid	PLSR	RAW	0.4614	0.9118	0.5989	0.8538
		SNV	0.4233	0.9257	0.5845	0.8607
		MSC	0.4232	0.9258	0.5968	0.8553
		SG smoothing	0.5249	0.8858	0.602	0.8509
		WT	0.4743	0.9067	0.5982	0.8538
	SVM	RAW	0.0631	0.9984	0.4328	0.9224
		SNV	0.0404	0.9993	0.2965	0.9636
		MSC	0.1035	0.9956	0.3016	0.9624
		SG smoothing	0.0839	0.9971	0.4278	0.9242
		WT	0.067	0.9982	0.4312	0.9229
	RF	RAW	0.2459	0.9687	0.5845	0.7854
		SNV	0.1735	0.9857	0.3954	0.9126
		MSC	0.1676	0.9866	0.3847	0.9168
		SG smoothing	0.2529	0.9674	0.6051	0.7727
		WT	0.2525	0.9673	0.5957	0.7782
Arachidic acid	PLSR	RAW	0.0927	0.8897	0.1219	0.8152
		SNV	0.0879	0.9008	0.1203	0.8186
		MSC	0.0874	0.9019	0.1221	0.8145
		SG smoothing	0.106	0.8557	0.1178	0.8261
		WT	0.0952	0.8838	0.1211	0.8174
	SVM	RAW	0.0762	0.9313	0.096	0.8848
		SNV	0.0204	0.9948	0.0577	0.9576
		MSC	0.0276	0.9907	0.0615	0.9526
		SG smoothing	0.0806	0.9245	0.0942	0.8905
		WT	0.077	0.93	0.0955	0.8864
	RF	RAW	0.0487	0.9587	0.0902	0.8161
		SNV	0.0317	0.9839	0.0548	0.9414
		MSC	0.0317	0.9843	0.0562	0.9421
		SG smoothing	0.0489	0.9592	0.0959	0.8029
		WT	0.0505	0.9554	0.0922	0.8123
Behenic acid	PLSR	RAW	0.1879	0.923	0.2517	0.8666
		SNV	0.176	0.9324	0.2485	0.8699
		MSC	0.1764	0.9321	0.2511	0.8678
		SG smoothing	0.214	0.9	0.2459	0.8701
		WT	0.1934	0.9184	0.2498	0.8679
	SVM	RAW	0.1229	0.969	0.208	0.9065
		SNV	0.0187	0.9992	0.1486	0.9521
		MSC	0.0184	0.9993	0.1543	0.9485
		SG smoothing	0.1334	0.9634	0.2091	0.9051
		WT	0.1244	0.9682	0.2078	0.9067
	RF	RAW	0.1026	0.9722	0.2312	0.8261
		SNV	0.0622	0.9905	0.1359	0.9486
		MSC	0.0589	0.9915	0.1347	0.9496
		SG smoothing	0.1012	0.9731	0.2348	0.8221
		WT	0.1038	0.9713	0.2293	0.8273

Table S4. Parameter setting of multivariate analysis methods using the full wavelengths

Fatty acids	Model	Pretreatment	Setting of parameters
Palmitic acid	PLSR	SNV	nLVs =13
		MSC	nLVs =14
	SVM	SNV	linear kernel, c =0.6, p = 0.01
		MSC	linear kernel, c = 4, p = 0.001
	RF	SNV	mtree=190, mtry= 53
		MSC	mtree=200, mtry= 50
Stearic acid	PLSR	SNV	nLVs =15
		MSC	nLVs =13
	SVM	SNV	linear kernel, c = 4, p = 0.01
		MSC	linear kernel, c = 5, p = 0.001
	RF	SNV	mtree=180, mtry= 58
		MSC	mtree=190, mtry= 52
Arachidic acid	PLSR	SNV	nLVs =14
		WT	nLVs =13
	SVM	SNV	linear kernel, c = 2, p = 0.001
		MSC	linear kernel, c = 0.5, p = 0.05
	RF	SNV	mtree=200, mtry= 44
		MSC	mtree=205, mtry= 48
Behenic acid	PLSR	SNV	nLVs =16
		SG smoothing	nLVs =12
	SVM	SNV	linear kernel, c = 1, p = 0.001
		MSC	linear kernel, c = 2, p = 0.001
	RF	SNV	mtree=195, mtry= 45
		MSC	mtree=200, mtry= 40

PLSR: nLVs - Number of PLS components. SVM: c - Penalty parameter C of the error term; p - Values of loss function in SVM. RF: ntree - Number of trees grown; mtry - Number of predictors sampled for splitting at each node.

Table S5. Parameter setting of multivariate analysis methods using the effective wavelengths

Fatty acids	Model	Setting of parameters
Palmitic acid	SNV+SPA+SVM	linear kernel, c = 10, p = 0.001
	SNV+VIP+SVM	linear kernel, c = 7, p = 0.09
	SNV+PCA+SVM	linear kernel, c = 12, p = 0.05
Stearic acid	SNV+SPA+SVM	linear kernel, c = 12, p = 0.0001
	SNV+VIP+SVM	linear kernel, c = 10, p = 0.01
	SNV+PCA+SVM	linear kernel, c = 15, p = 0.0001
Arachidic acid	SNV+SPA+SVM	linear kernel, c = 10, p = 0.001
	SNV+VIP+SVM	linear kernel, c = 10, p = 0.01
	SNV+PCA+RF	mtree= 100, mtry= 3
Behenic acid	SNV+SPA+RF	mtree= 100, mtry= 4
	SNV+VIP+SVM	linear kernel, c = 5, p = 0.01
	SNV+PCA+RF	mtree= 100, mtry=2

SVM: c - Penalty parameter C of the error term; p - Values of loss function in SVM. RF: ntree - Number of trees grown; mtry - Number of predictors sampled for splitting at each node.
